# ICTV Virus Taxonomy Profile: *Arenaviridae*

**DOI:** 10.1099/jgv.0.001280

**Published:** 2019-06-13

**Authors:** Sheli R. Radoshitzky, Michael J. Buchmeier, Rémi N. Charrel, J. Christopher S. Clegg, Jean-Paul J. Gonzalez, Stephan Günther, Jussi Hepojoki, Jens H. Kuhn, Igor S. Lukashevich, Víctor Romanowski, Maria S. Salvato, Manuela Sironi, Mark D. Stenglein, Juan Carlos de la Torre, ICTV Report Consortium

**Affiliations:** 1 United States Army Medical Research Institute of Infectious Diseases, Fort Detrick, Frederick, MD 21702, USA; 2 Department of Molecular Biology and Biochemistry, University of California, Irvine, CA 92697, USA; 3 Unité des Virus Emergents, Aix-Marseille Univ, Marseille, France; 4 Les Mandinaux, 16450 Le Grand Madieu, France; 5 Center of Excellence for Emerging and Zoonotic Animal Disease, Kansas State University, Manhattan, KS 66502, USA; 6 Department of Virology, Bernhard Nocht Institute for Tropical Medicine, 20359 Hamburg, Germany; 7 Department of Virology, Medicum, University of Helsinki, Helsinki, Finland; 8 Institute of Veterinary Pathology, University of Zurich, Zurich, Switzerland; 9 NIH/NIAID/IRF-Frederick, Fort Detrick, MD 21702, USA; 10 Department of Pharmacology and Toxicology, School of Medicine, and the Center for Predictive Medicine for Biodefense and Emerging Infectious Diseases, University of Louisville, Louisville, KY 40202, USA; 11 IBBM, Facultad de Ciencias Exactas, Universidad Nacional de La Plata - CONICET, La Plata, Argentina; 12 Institute of Human Virology, University of Maryland School of Medicine,, Baltimore, MD 21201, USA; 13 Bioinformatics, Scientific Institute IRCCS E. MEDEA, 23842 Bosisio Parini, Italy; 14 Department of Microbiology, Immunology and Pathology, Colorado State University, Fort Collins, CO 80523, USA; 15 Department of Immunology and Microbiology IMM-6, The Scripps Research Institute, La Jolla, CA 92037, USA

**Keywords:** *Arenaviridae*, arenavirus, ICTV Report, mammarenavirus, reptarenavirus, taxonomy

## Abstract

Members of the family *Arenaviridae* produce enveloped virions containing genomes consisting of two or three single-stranded RNA segments totalling about 10.5 kb. Arenaviruses can infect mammals, including humans and other primates, snakes, and fish. This is a summary of the International Committee on Taxonomy of Viruses (ICTV) Report on the family *Arenaviridae*, which is available at www.ictv.global/report/arenaviridae.

## Virion

**Fig. 1.**
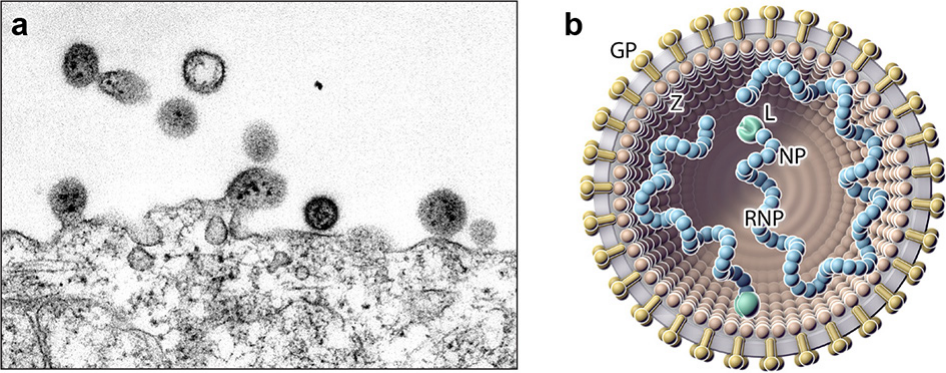
(a) Electron micrograph of lymphocytic choriomeningitis virus particles, showing dark internal inclusion bodies, budding from an infected cell. (**b**) Schematic illustration of a (mammalian) arenavirus particle. Shown is the spherical and enveloped (grey) particle that is spiked with glycoproteins (GP, gold) around a layer of zinc finger matrix proteins (Z, brown; missing in hartmaniviruses). The small and large ribonucleoprotein (RNP) complexes inside the particle consist of nucleoprotein (NP, blue) and RNA-dependent RNA polymerase (L, green).

Virions are spherical or pleomorphic in shape, 40–200 nm in diameter, with dense lipid envelopes (Table 1 and Fig. 1). The virion surface layer is covered with club-shaped projections with distinctive stalk and head regions. These projections consist of trimeric spike structures of two virus-encoded membrane glycoprotein (GP) subunits (GP1 and GP2) and, in the case of some arenaviruses, a stable signal peptide (SSP). Isolated ribonucleoprotein (RNP) complexes appear as ‘beads-on-a-string’-like structures [1–3].

**Table 1.** Characteristics of members of the family *Arenaviridae*

Typical member:	lymphocytic choriomeningitis virus, Armstrong 53b (S segment: AY847350; L segment: AY847351), species *Lymphocytic choriomeningitis mammarenavirus*, genus *Mammarenavirus*
Virion	Enveloped, pleomorphic virions 40–200 nm in diameter with trimeric surface spikes
Genome	Two or three single-stranded, usually ambisense, RNA molecules called small (S), medium (M) and large (L)
Replication	Ribonucleoprotein complexes containing anti-genomic RNA serve as templates for synthesis of genomic RNA
Translation	From capped and non-polyadenylated mRNAs. The 5′-cap structure is derived by polymerase slippage or cap-snatching from cellular mRNAs
Host range	Fish (antennaviruses), mammals (mammarenaviruses) and reptiles (hartmaniviruses and reptarenaviruses), but possibly also bats and ticks
Taxonomy	Realm *Riboviria,* phylum *Negarnaviricota*, subphylum *Polyploviricotina*, class *Ellioviricetes*, order *Bunyavirales*. The family includes several genera and >40 species

## Genome

**Fig. 2.**
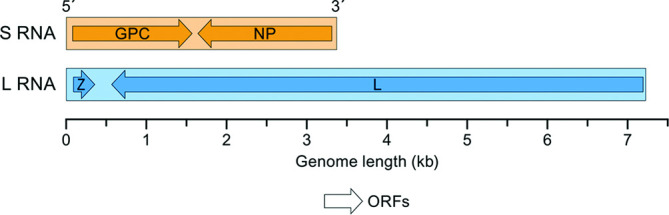
Schematic representation of the bisegmented genome organization of the mammalian arenavirus lymphocytic choriomeningitis virus. The 5′- and 3′-ends of both segments (**S and L**) are complementary at their termini, probably promoting the formation of circular RNP complexes within the virion. GPC, glycoprotein precursor; L, RNA-dependent RNA polymerase; NP, nucleoprotein; Z, zinc finger matrix protein. ORFs are separated by non-coding IGRs with predicted hairpin structures (not shown).

Arenavirus genomes consist of two or three single-stranded, typically ambisense RNA molecules, termed small (S), medium (M) and large (L). Some of these RNAs encode two proteins in non-overlapping ORFs of opposite polarities that are separated by non-coding intergenic regions (IGRs) (Fig. 2). The S RNA encodes nucleoprotein (NP) in the virus genome-complementary sequence, and, in many cases, the virus glycoprotein precursor (GPC) in the virus genome-sense sequence. The L RNA encodes the L protein in the virus genome-complementary sequence, and, in some cases, the zinc-finger matrix protein (Z) in the virus genome-sense sequence [1–4].

## Replication

Arenaviruses attach to cell-surface receptors and enter via the endosomal route. pH-dependent fusion with late endosomes releases the virion RNP complex into the cytoplasm. In some arenaviruses, this pH-dependent fusion event requires the previous participation of an intracellular receptor. The virus RNP directs both RNA genome replication and gene transcription. During replication, L reads through the IGR transcription-termination signal and generates uncapped antigenomic and genomic RNAs. In ambisense coding arrangements, transcription of mRNAs encoding GPC and Z occurs only after the first round of virus replication, during which S and L antigenomes are produced.

Arenavirus proteins are synthesized from subgenomic mRNAs that lack 3′-terminal poly(A) and in which the 5′-cap is followed by several non-templated bases, possibly the result of cap-snatching.

Virion budding occurs from the cellular plasma membrane, thereby providing the virion envelope [1–3].

## Taxonomy

Arenaviruses form a family in the order *Bunyavirales*. Within this order, arenaviruses are most closely related to members of the family *Mypoviridae*. Arenaviruses differ from most other bunyaviruses by having segmented genomes with an ambisense organization. The family includes several genera and >40 species. Some arenaviruses can cause severe and sometimes fatal diseases in humans (e.g. Lassa fever) [5]. Other arenaviruses cause disease in captive snakes [4, 6], and some arenaviruses can infect fish [4].

### Resources

Full ICTV Report on the family *Arenaviridae*: www.ictv.global/report/arenaviridae.

## Abbreviations

GP: glycoprotein
GPC: glycoprotein precursor
IGR: intergenic region
NP: nucleoprotein
RNP: ribonucleoprotein
SSP: stable signal peptide.
